# Improvement of alignment accuracy utilizing sequentially conserved motifs

**DOI:** 10.1186/1471-2105-5-167

**Published:** 2004-10-28

**Authors:** Saikat Chakrabarti, Nitin Bhardwaj, Prem A Anand, Ramanathan Sowdhamini

**Affiliations:** 1 National Centre for Biological Sciences (TIFR), Bangalore 560065, India; 2 Department of Chemical Engineering, Indian Institute of Technology, Mumbai, India; 3 International Institute of Information Technology (MSIT), Hyderabad, India

## Abstract

**Background:**

Multiple sequence alignment algorithms are very important tools in molecular biology today. Accurate alignment of proteins is central to several areas such as homology modelling, docking studies, understanding evolutionary trends and study of structure-function relationships. In recent times, improvement of existing progressing programs and implementation of new iterative algorithms have made a significant change in this field.

**Results:**

We report an alignment algorithm that combines progressive dynamic algorithm, local substructure alignment and iterative refinement to achieve an improved, user-interactive tool. Large-scale benchmarking studies show that this FMALIGN server produces alignments that, aside from preservation of functional and structural conservation, have accuracy comparable to other popular multiple alignment programs.

**Conclusions:**

The FMALIGN server allows the user to fix conserved regions in equivalent position in the alignment thereby reducing the chance of global misalignment to a great extent. FMALIGN is available at

## Background

The advent of large genome projects has led to an explosion of sequence data in public databases. Analysis of protein families, understanding their evolutionary trends and detection of remote homologues are now the primary objectives. Genome annotation and analysis tools like fold prediction, homology modelling, protein-ligand docking and clustering algorithms rely heavily on accurate multiple alignments to provide a genome-wide perspective.

The most popular approach for multiple sequence alignment has been the progressive alignment method [1]. A multiple alignment is built up gradually by aligning the closest sequences first and successively adding in more distant relatives. A number of alignment programs imply this algorithm, for example MULTALIGN [2], MULTAL [3] and CLUSTALX [4]. They employ a global alignment algorithm to construct an alignment over the entire length of the sequences and differ mainly in the procedure employed to determine the order of alignment of the sequences. The most common usage is the sequential branching method to identify two closest sequences first and subsequently align the next closest sequence to those already aligned. MULTALIGN [2] constructs a guided tree using UPGMA [5] method. A consensus method is then used to align larger and larger groups of sequences according to the branching order of the tree. CLUSTALX uses the alternative neighbour-joining algorithm [6] to construct the tree. In contrast to the above global method, PIMA [7] uses a local dynamic programming algorithm to align only the most conserved motifs. In addition, numerous new alignment algorithms have recently been developed which offer fresh approaches to the multiple alignment problem. A common point of interest has been the application of iterative strategies to refine and improve the initial alignment. A local alignment approach is implemented in the DIALIGN program [8,9] to construct multiple alignments based on segment-to-segment comparison rather than residue-to-residue comparison using an iterative strategy to improve alignment accuracy. Alignment programs like MATCH-BOX [10] utilize statistical similarity measures to delineate sequentially conserved regions and the final alignments are derived by those of the conserved "box" regions. The regions outside the "boxes" are not aligned. METAMEME [11] is a motif based search engine that aligns motif regions found in the target sequences. DbClustal [12] combines the advantages of both local and global alignment algorithm in a traditional tree-based progressive alignment. Starting from ClustalW [22], which is a global alignment program, local alignment data or anchor points are incorporated in the dataset. The global alignment is then weighted towards, but not constrained, to the locally conserved segments and the alignment is not subject to iterative refinements. MACAW [13] provides a user interactive interface to select conserved segments from the alignment but these segments are not utilized further to refine the resulting alignment.

In this paper, we present an alignment algorithm that combines the properties of both progressive alignment methods and iterative refinement algorithms. This algorithm offers the user the selective advantage of guiding the course of alignment by simultaneous inputs of multiple conserved motif regions that in turn guarantees retention of structure/function in the final alignment. FMALIGN is an alignment server that provides the user to obtain a control over the alignment by providing important conserved regions as input to the alignment program to achieve a more structurally relevant and functionally useful alignment of protein sequences. It employs the sequential branching method to identify the closest pair of sequences and subsequently includes the next closest sequences to generate a guided tree using UPGMA [5], which in turn dictates the sequential order of the alignment. FMALIGN also considers the local similarity of the sequences in the conserved motif regions; as the name implies, it allows local conserved regions of the sequences to be fixed and aligns the rest based on normal progressive alignment. The chances of global misalignment are thereby reduced and the possibility of obtaining overall better alignment is increased. The FMALIGN server also offers an iterative refinement option where a routine (FINDMOTIF) identifies more conserved regions in the derived alignment and allows the user to provide fresh 'equivalences' to obtain an overall better alignment. Benchmarking studies on difficult alignments, examined in BAliBASE [14], show promising prospect for the FMALIGN server to be an useful alignment algorithm.

### Implementation: methodology and description of FMALIGN server

**Figure 1:**
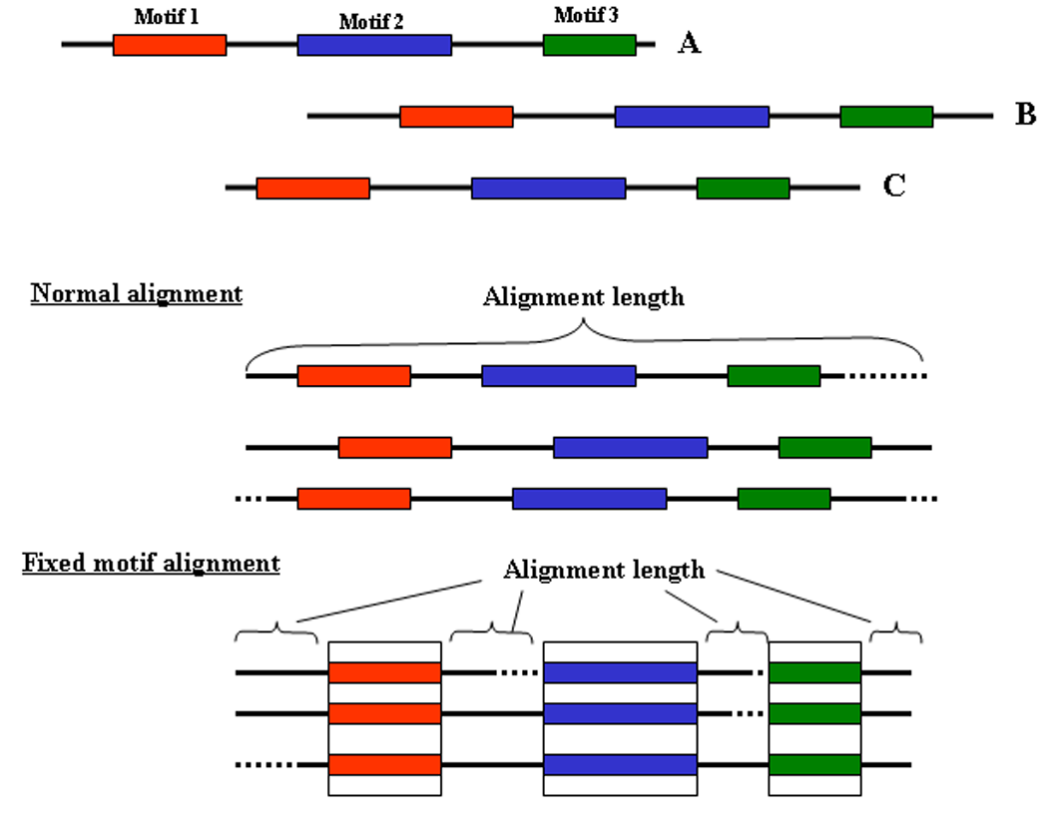
Cartoon representation of the flowchart of FMALIGN methodology. Coloured parts are meant to be important segments of protein sequences.

The algorithm of FMALIGN (Fixed Motif ALIGNment) program combines three main criteria: (a) Progressive global alignment method, (b) substructure (local) conserved region fixation and (c) iterative refinement of alignment with identification of more conserved regions. The procedure involves three steps: (i) identification and fixing of the specified sequential conserved regions. This alignment method requires specific regions of the sequences to be aligned as anchor and these anchors are generally meant for sequentially conserved parts which do not undergo many changes. (ii) derivation of the progressive multiple sequence alignment guided by the tree. During this step, excluding the fixed anchored regions, the rest of the sequences are divided into several sub-segments that are aligned employing a dynamic alignment algorithm in a sequential order from N to C-terminus. The phylogenetic guide tree has been derived using Unweighted Pair Group Method with Arithmetic Mean (UPGMA) [5] which is the simplest method of tree construction. The UPGMA method was adopted since the whole length of the protein is divided into several segments considering them as ultrametric After all the sub-segment alignments are performed, the aligned parts as well as the selected, fixed motif regions are combined to produce the full-length alignment for a group of proteins under the hierarchical cluster. This process continues until all the protein sequences are aligned multiply. (iii) The iterative refinement of alignment subsequent to the identification of more motifs. In this step, more conserved regions are identified by observing the amino acid exchanges in the resultant alignment derived from the previous iteration. These conserved regions are then used as anchors together with previously identified motifs and the whole process is repeated until an optimal alignment having maximum number of conserved regions and alignment score is obtained. The algorithm thus combines the progressive dynamic algorithm for global multiple alignment and selected conserved regions or local alignments. Once the primary alignment is derived, the second step can be repeated by including more conserved regions from this alignment as motifs to derive a better alignment. Figure 1 shows a cartoon representation of the methods in a flowchart diagram.

FMALIGN server can accept amino acid patterns for multiple motifs provided by the user. It also provides option to the user to search motifs within their sequences using FINDMOTIF routine or to obtain them for the alignment by providing a link to SMoS [15], a structural motif database for aligned protein superfamilies. The FINDMOTIF routine in the server provides sequential conserved regions for a set of proteins on the basis of sequence similarity and a 20 × 20 substitution matrix by consulting large number of structure-based sequence alignments of homologous families [16]. Amino acid exchange scores at every alignment position are assigned the same as the element in this matrix for all possible pairs of proteins and averaged over the number of pairs. Contiguous alignment positions with an average amino acid exchange score over 50 (for homologues) or 40 (for superfamilies) are recognised as motifs. FMALIGN also offers an option for the user to refine the derived alignment by generating more sequential conserved regions through FINDMOTIF option. The inter-motif regions are aligned by normal progressive alignment using standard substitution matrices like BLOSUM62. The gap penalties used in this version of FMALIGN are all maintained according to standard multiple alignment parameters.

#### Alignment scores

To assess the performance of FMALIGN in comparison to other programs, Sum-of-Pair-score (SPS) and Column-Score (CS) alignment scoring scheme [14] are applied on FMALIGN derived alignments to assess the quality of alignments compared to BAliBASE reference alignments. SPS and CS are calculated such that the score increases with the number of sequences aligned accurately and is used to determine the extent to which the programs succeed in aligning some, if not all, of the sequences in an alignment correctly [14]. The scores used to measure the performance of the various alignment programs may not be appropriate for all the datasets. Therefore, for each reference test the most suitable scoring function have been selected according to the nature of the benchmarking.

## Results and discussion

### Benchmarking at family level

**Figure 2:**
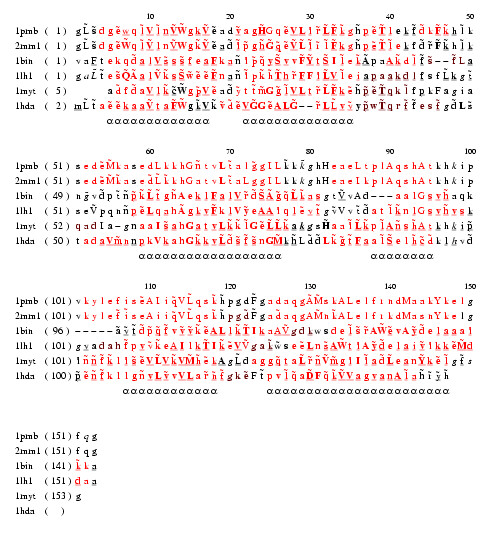
Six members from globin family are selected and aligned by FMALIGN utilizing sequentially conserved regions. The structure-annotated JOY [24] representation of the alignment shows high conservation of structural features retained in this alignment. Capital and small letters represent solvent-buried and solvent-exposed residues, respectively. Helices and β-strands are represented in red and blue colours, respectively. Concensus secondary structures are marked. Hydrogen bonding patterns are also annotated: hydrogen bond to main chain and side chain are indicated in bold and underlined, respectively. Residues with positive-φ conformation are italised.

FMALIGN derived alignments retain high degree of conservation in secondary structures. Six members from the globin family were selected comprising a wide range of sequence identity between them (7% to 61%). Conserved regions for six aligned globin sequences were identified by the FINDMOTIF routine starting from CLUSTALX [4] alignment with default parameters and aligned by the FMALIGN server. The resultant alignment shows high degree of secondary structural conservation despite the difficulty of aligning a set of sequences having very wide range of sequence identity (Figure 2).

In order to evaluate the FMALIGN server, the results have been compared with 10 other alignment programs and objective criteria were employed to assess the quality of an alignment. We selected the BAliBASE benchmark alignment database [14] to compare the performance of the FMALIGN alignment server. The BAliBASE benchmark alignment database contains 142 reference alignments, divided into five reference sets each containing at least 12 representative alignments. Performance of FMALIGN is checked on all five reference sets provided in BAliBASE datasets.

Reference 1 alignments consist of a small number of equidistant sequences of similar length, i.e. the percent residue identity (% ID) between any two sequences is within a specified range and no large extensions or insertions have been introduced.

Reference 2 contains alignments of a family of closely related sequences with >25% ID, plus up to three 'orphan' sequences (distant members of the family with <20% ID, sharing a common fold). It is designed to evaluate program accuracy according to two criteria: (i) the stability of the family alignment when orphans are introduced into the sequence set and (ii)the quality of the alignment of the orphan sequences.

Reference 3 checks the ability of the programs to correctly align equidistant divergent families into a single alignment. The reference alignments consist of up to four families, with <25% ID between any two sequences from different families.

Reference 4 and 5 contain alignments of upto 20 sequences including N/C-terminal extensions (upto 400 residues) and insertions (upto 100 residues), respectively.

#### Reference 1: a small number of approximately equidistant sequences

**Figure 3:**
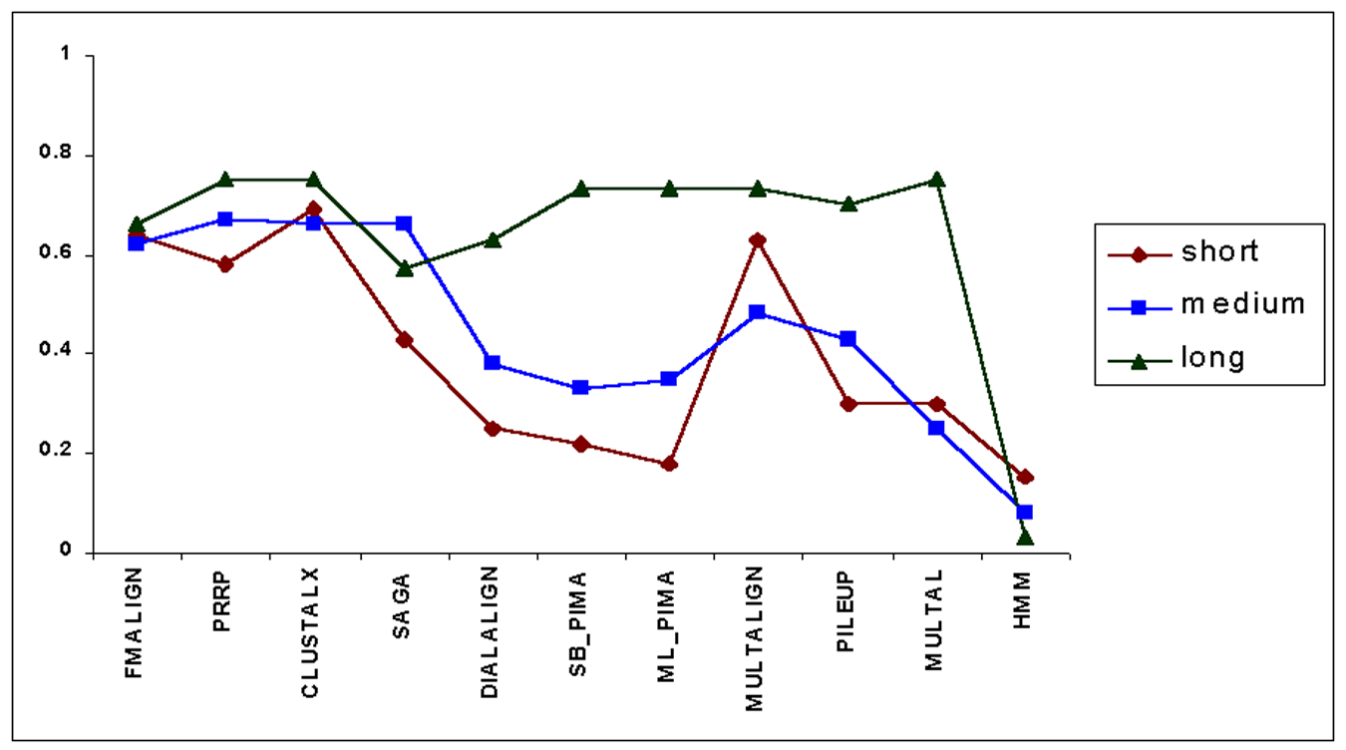
SPS for reference 1 for all three categories. a) SPS scores are shown for VI (<25% sequence identity) (b) for V2 (20%–40% sequence identity), and (c) V3 (>35% sequence identity). Alignments of different length categories are shown by different line plot.

**Figure 4:**
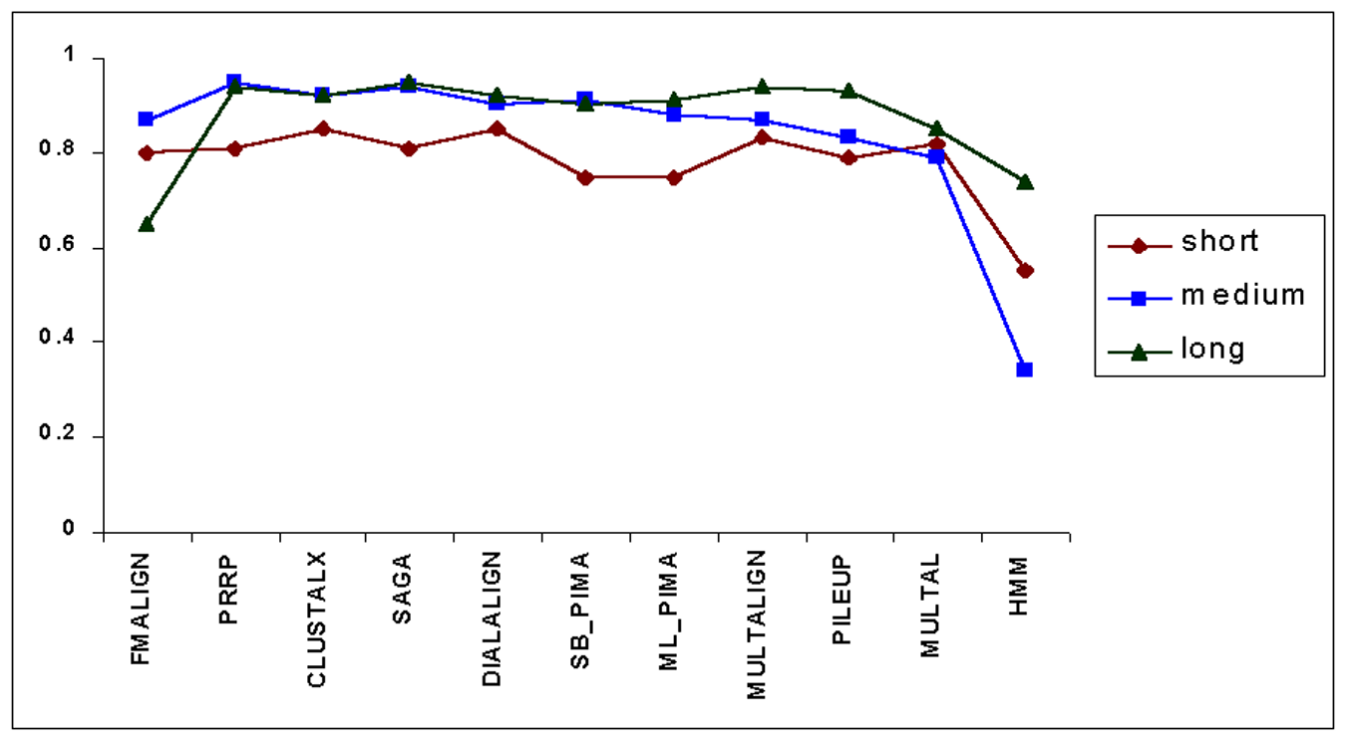
SPS scores for reference 1 for V2 (20% -40% sequence identity). Alignments of different length categories are shown by different line plot.

**Figure 5:**
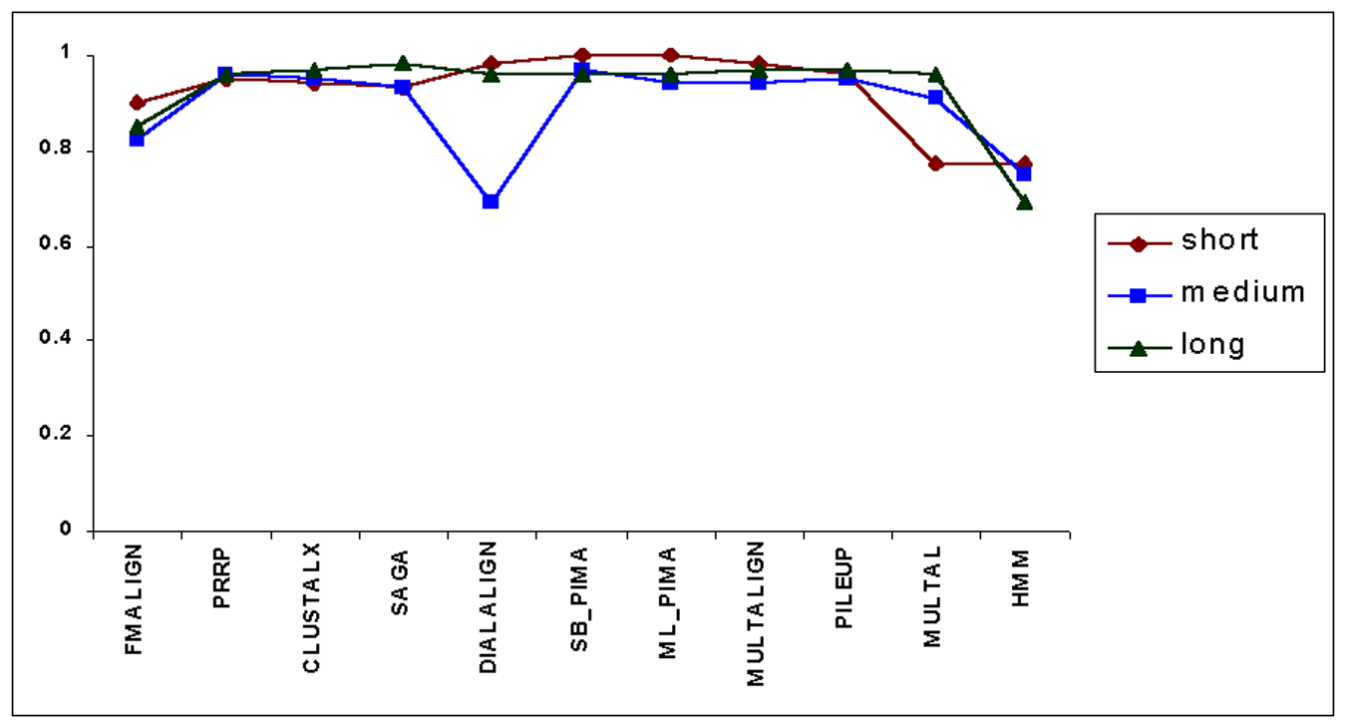
SPS scores for reference 1 for V3 (>35% sequence identity). Alignments of different length categories are shown by different line plot.

This dataset is designed to study the effect of sequence length and percentage identity on the performance of the alignment program and provides a basis for the remaining tests. The overall performance of FMALIGN server for this dataset is comparable to the two best performing alignment programs, like PRRP [17] and CLUSTALX [4], in all three categories (VI, V2, and V3) based on sequence identity and alignment lengths (short, medium and long) as shown in Figure 3, 4, 5.

#### Reference 2: a related family with divergent, orphan sequences

**Figure 6:**
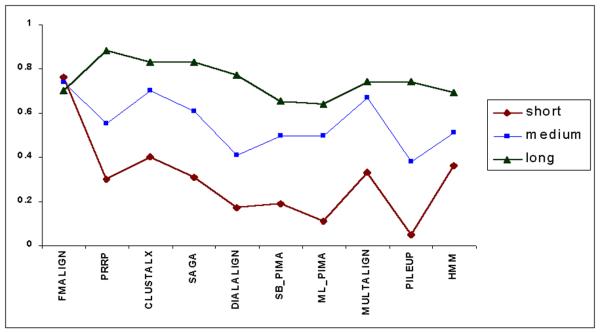
Alignment scores (SPS) are compared for reference 2. Alignments of different length categories are shown by different line in the plot.

It is possible to assess the performance of the methods to align divergent 'orphan' sequences (10–20% ID with the family and between orphans) with a family of highly related (>25% ID) sequences using this data set. It is also interesting to observe the disruption of the family alignment due to the introduction of orphans. Figure 6 shows SPS for the alignment of a single orphan against a closely related family. The global alignment programs again perform better than the local ones in this test. However, CLUSTALX and SAGA [18] now rank above PRRP. The performance of FMALIGN server is significantly better than other programs for all the three length categories.

#### Reference 3: families of related sequences

**Figure 7:**
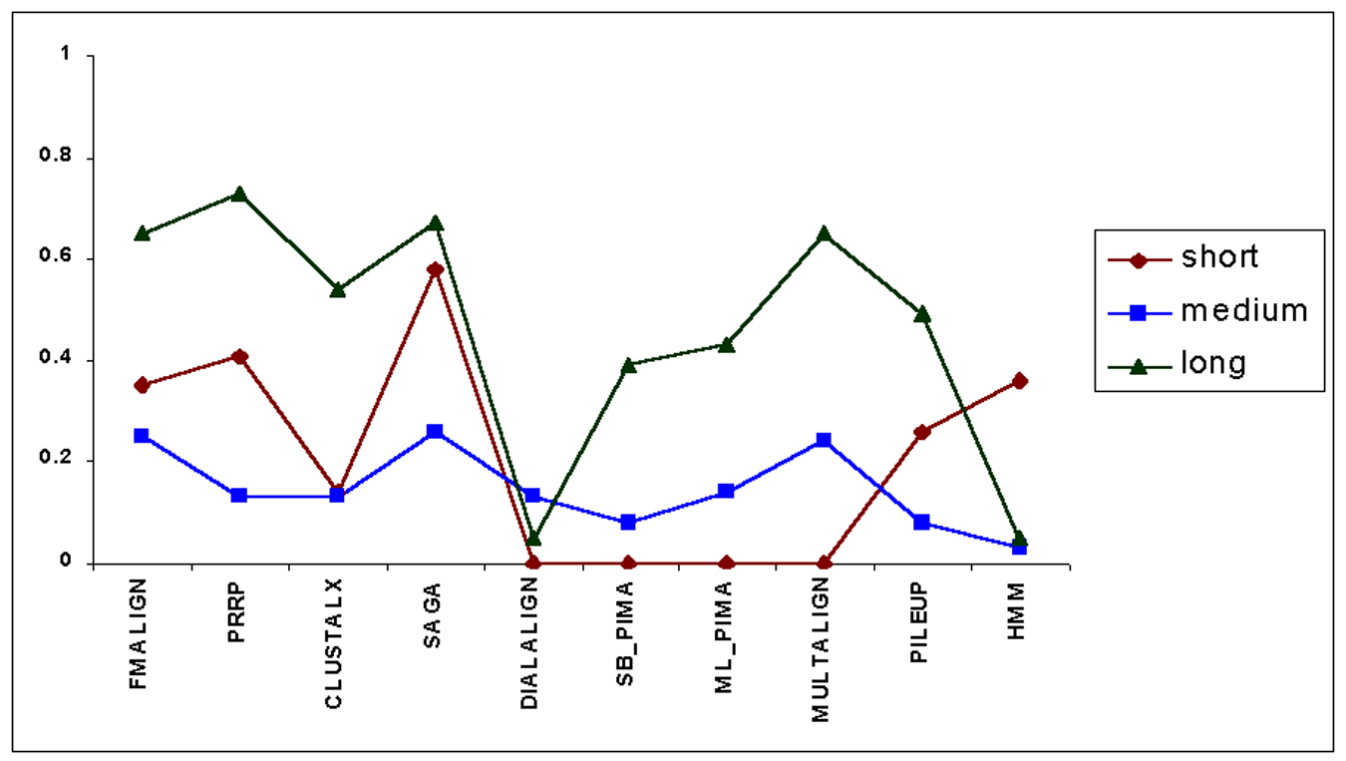
Alignment scores (CS) are compared for reference 3. Alignments of different length categories are shown by different line in the plot.

This allows the assessment of the programs to correctly align approximately equidistant divergent families (<20% ID) composed of highly related sequences (>25% ID) into a single multiple alignment. Figure 7 shows the scores for the programs in the order. The iterative strategies of PRRP [17] and SAGA [18] perform better in this test than the traditional progressive alignment methods. However, FMALIGN performs better than the other progressive methods, with the global methods generally ranking higher than the local methods.

#### Reference 4: N/C-terminal extensions

**Figure 8:**
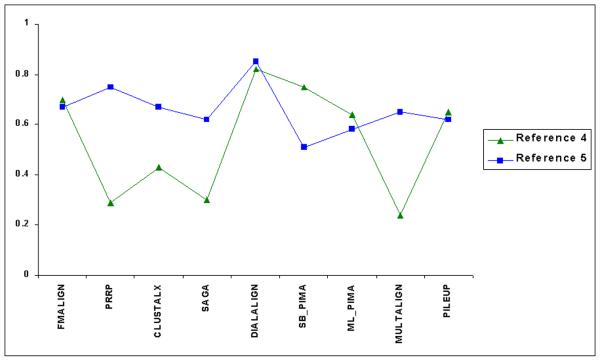
Alignment scores (CS) are compared for reference 4 and 5.

This dataset includes large N/C-terminal extensions to investigate whether the programs are capable of aligning the core blocks flanking the extensions. No large internal insertions are introduced at this stage. Mostly local alignment strategies out-perform the global methods. PILEUP (Wisconsin Package v.8; Genetics Computer Group, Madison, WI) is the only program based on a global alignment method which does reasonably well compared to other global methods. Performance of FMALIGN is comparable to the best three methods (DIALIGN [8], SB_PIMA [19] and PILEUP) as shown in Figure 8.

#### Reference 5: internal insertions

In contrast to reference 4, in this dataset the insertions are internal to the homologous domains and not at the N/C-terminus as overhangs. FMALIGN also performs well in this category and results are comparable to the other better performing global alignment methods like PRRP and CLUSTALX as shown in Figure 8.

### Benchmarking at superfamily level

#### Utilization of structural motifs to improve alignment accuracy

**Figure 9:**
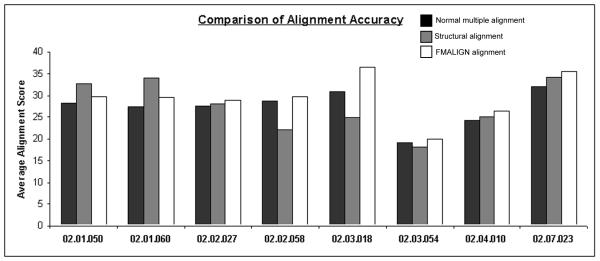
Comparison of alignment accuracy on the basis of sequence similarity. Conserved motifs are identified for superfamilies like, Cytochromes (code: 02.01.050), ACP-like (02.01.060), C2 domain (Calcium/lipid-binding domain, CaLB) (code: 02.02.027), ISP domain (code: 02.02.058), Phosphatidylinositol-specific phospholipase C (PI-PLC) (code: 02.03.018), Esterase/acetylhydrolase (02.03.054), Chromo domain-like (code: 02.04.010) and BPTI-like (02.07.023). These motifs are utilized to realign the sequences by FMALIGN. Alignments are scored using sequence similarity scores based on a 20x20 substitution matrix. FMALIGN derived alignments are compared against CLUSTALX [4] and sequence-structure alignment, COMPARER [21].

The performance of FMALIGN has been tested on a dataset of representative superfamilies of proteins belonging to different structural classes in PASS2 [20] and SMoS [15] databases. The structural motifs from the SMoS database have been utilized as conserved regions for the member of the superfamily (average sequence identity less than 30%). All the proteins of a superfamily have been aligned. Each alignment is assigned a quality score by averaging the amino acid exchange score of each column over the length of the alignment (see method for details). The alignments derived from FMALIGN have been compared against the CLUSTALX-derived sequence alignment as well as the sequence-structure alignment derived from COMPARER [21]. Figure 9 shows an equivalent or better accuracy of the FMALIGN server compared to CLUSTALX and COMPARER-derived alignment. This indicates that FMALIGN is particularly efficient for specific sets of sequences for which the degree of conservation is known. The initial results also indicate that FMALIGN can perform very well and provide an alignment which is very similar or better than a structurally derived alignment.

#### Utilization of sequence motifs to improve alignment accuracy

**Figure 10:**
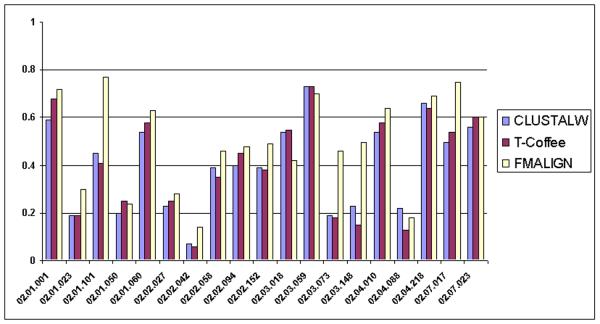
Comparison of alignment accuracy. FMALIGN derived alignments of the PASS2 [20] superfamily members are compared against the same derived by CLUSTALW [22] and T-Coffee [23].

Representative superfamily alignments belonging to different structural classes from the PASS2 [20] superfamily alignment database are taken for a benchmarking test (Table 1). These superfamily alignments are of different lengths (short, medium, and long) and possess an average sequence identity that ranges from 10% to 26%. Sequential conserved regions or motifs are identified for each superfamily alignment and utilized in the FMALIGN server to realign the superfamily members. Similarly, the superfamily members are also aligned by the two best performing multiple alignment methods, CLUSTALW [22] and T-Coffee [23]. These alignments are then compared against the structure-based COMPARER alignments provided by PASS2 database using the same alignment scoring scheme (Sum-of-Pair score) of the BAliBASE benchmarking database. FMALIGN-derived alignments performed better for most of the cases compared to the other two methods as shown in Figure 10.

## Conclusions

FMALIGN server provides a web interface for pairwise and multiple sequence alignments of proteins. FMALIGN provides an alignment by combining progressive dynamic algorithm, local substructure alignment and iterative refinement presenting an improved, user interactive alignment procedure. FMALIGN server allows the user to fix conserved regions in equivalent positions amongst the sequences to be aligned leading to alignments that are reliable and biologically more meaningful. Additional options for the users to choose substitution matrices and gap penalty values may be incorporated in future.

All the alignments provided in BAliBASE are realigned by FMALIGN and the resulting scores (both SPS and CS) are calculated using a standard program kindly provided by the authors of BAliBASE. The inter-motif regions are aligned in FMALIGN by normal progressive alignment using standard substitution matrices like BLOSUM62. The gap penalties used in this version of FMALIGN are all maintained according to standard multiple alignment parameters. Benchmarking at the superfamily level has also been done utilizing both structural and sequential conserved regions. The dataset is wide enough to include proteins from different structural classes and of different length. The average sequence identity for this dataset was less than 30% since the proteins are related at the superfamily level. The performance of FMALIGN has been tested against one of the best performing multiple sequence alignment (T-Coffee) as well as structure based alignment tool (COMPARER). Studies on large and different datasets revealed an overall better performance of FMALIGN server for all categories in BAliBASE benchmarking database. It works especially well at lower sequence identity range, such as superfamily level, where no two proteins are more than 25% identical to each other in comparison to other popular methods like CLUSTALX and T-Coffee. It is well-known that automatic multiple sequence alignments at poor sequence identity are often subject to careful manual validations and improvements to avoid offsets of critical functionally important residues. FMALIGN can sensitively address this issue to avoid manual intervention subsequent to final alignment. FMALIGN-derived alignments also show a high conservation of secondary structural elements and provide better alignments for comparative modelling. The implementation of this alignment algorithm can be used to include new members into an existing protein superfamily with the help of motif regions that provide a reliable approach to connect protein sequences with their structural homologues within a particular superfamily. FMALIGN is available via the following URL 

## Authors' contributions

S.C. had carried out the benchmarking, was involved in the development of the server and has written the first draft of the manuscript. N.B. had written the initial part of the code and P.A. has written the latter part of the code and developed a web server. R.S. had initiated the idea, was involved in discussions and in the critical reading of the manuscript.

## List of abbreviations

FMALIGN Fixed Motif ALIGNment

SPS Sum-of-Pairs Score

## Figures and Tables

**Table 1:** List of superfamilies used for FMALIGN benchmarking.

**Superfamily code**	**Superfamily name**	**Structural Class**	**Average length of proteins**	**Average sequence identity(%)**
02.01.001	Globin-like	All α	142	15.2
02.01.023	Putative DNA-binding domain	All α	76	10.0
02.01.050	Cytochromes	All α	122	22.0
02.01.060	ACP-like	All α	80	23.5
02.01.101	SAM/Pointed domain	All α	84	13.3
02.02.027	C2 domain(Calcium/lipid-binding domain, CaLB)	All β	75	11.4
02.02.042	Galactose-binding domain-like	All β	185	12.6
02.02.058	ISP domain	All β	136	23.4
02.02.094	Acid proteases	All β	217	20.4
02.02.152	Hedgehog/intein (Hint) domain	All β	177	16.9
02.03.018	Phosphatidylinositol-specific phospholipase C (PI-PLC)	α and β	286	13.6
02.03.059	Ferredoxin reductase-like, C-terminal NADP-linked domain	α and β	132	21.6
02.03.073	Thiamin diphosphate-binding fold (THDP-binding)	α and β	236	12.1
02.03.148	"Helical backbone" metal receptor	α and β	320	14.4
02.04.010	Chromo domain-like	α and β	68	26.3
02.04.088	Regulatory domain in the amino acid metabolism	α and β	90	12.2
02.04.218	Ribosome inactivating proteins (RIP)	α and β	253	22.1
02.07.017	Leech antihemostatic proteins	α and β	47	19.1
